# Similarities and differences of functional connectivity in drug-naïve, first-episode adolescent and young adult with major depressive disorder and schizophrenia

**DOI:** 10.1038/srep44316

**Published:** 2017-03-13

**Authors:** Shengnan Wei, Fay Womer, Haiyang Geng, Xiaowei Jiang, Qian Zhou, Miao Chang, Yifang Zhou, Yanqing Tang, Fei Wang

**Affiliations:** 1 Brain Function Research Section, First Affiliated Hospital, China Medical University, Shenyang, Liaoning, PR China; 2 Department of Radiology, First Affiliated Hospital, China Medical University, Shenyang, Liaoning, PR China; 3 Department of Psychiatry, Washington University School of Medicine, St. Louis, Missouri, USA; 4 Department of Psychiatry, First Affiliated Hospital, China Medical University, Shenyang, Liaoning, PR China; 5 Department of Geriatric Medicine, First Affiliated Hospital, China Medical University, Shenyang, Liaoning, PR China; 6 Department of Psychiatry, Yale University School of Medicine, New Haven, Conn., USA

## Abstract

Major depressive disorder (MDD) and schizophrenia (SZ) are considered two distinct psychiatric disorders. Yet, they have considerable overlap in symptomatology and clinical features, particularly in the initial phases of illness. The amygdala and prefrontal cortex (PFC) appear to have critical roles in these disorders; however, abnormalities appear to manifest differently. In our study forty-nine drug-naïve, first-episode MDD, 45 drug-naïve, first-episode SZ, and 50 healthy control (HC) participants from 13 to 30 years old underwent resting-state functional magnetic resonance imaging. Functional connectivity (FC) between the amygdala and PFC was compared among the three groups. Significant differences in FC were observed between the amygdala and ventral PFC (VPFC), dorsolateral PFC (DLPFC), and dorsal anterior cingulated cortex (dACC) among the three groups. Further analyses demonstrated that MDD showed decreased amygdala-VPFC FC and SZ had reductions in amygdala-dACC FC. Both the diagnostic groups had significantly decreased amygdala-DLPFC FC. These indicate abnormalities in amygdala-PFC FC and further support the importance of the interaction between the amygdala and PFC in adolescents and young adults with these disorders. Additionally, the alterations in amygdala-PFC FC may underlie the initial similarities observed between MDD and SZ and suggest potential markers of differentiation between the disorders at first onset.

Psychiatric diagnosis is challenging, particularly during the initial phases of illness. As a field, we still rely heavily on longitudinal observation for definitive diagnosis, often resulting in considerable delay in appropriate diagnosis and treatment. With converging evidence of significant neural abnormalities by the time of illness onset, early identification and intervention is increasingly critical in efforts to substantially alter the trajectory of psychiatric illnesses such as schizophrenia (SZ) and major depressive disorder (MDD). MDD and SZ are severely disabling psychiatric disorders that have long been conceptualized as two distinct illnesses. However, MDD and SZ have considerable overlap in behavioral manifestations and impairments in emotional processing and cognitive functioning^1^,^2^,^3^, particularly during their initial phases^4^,^5^,^6^,^7^,^8^. Frequently, prodromal SZ resembles a major depressive episode and psychotic symptoms are present in first episode MDD, complicating differentiation between the two disorders at initial onset^5^. In first episode psychosis, significant negative symptoms, which traditionally has been viewed as hallmarks of SZ, have been observed in both SZ and MDD, as well as depressive symptoms in first episode SZ^4^,^5^. These overlaps in presentation at initial onset challenge effective treatment interventions during the early course of these disorders and ultimately the potential for marked improvement in clinical outcomes. Treatment approaches toward MDD and SZ differ in targets, pharmacologic agents, use of somatic therapy, and duration of treatment, and hence accurate diagnosis is important in determining appropriate treatment for individuals with these disorders. Neural biomarkers could vastly improve differentiation between psychiatric disorders such as MDD and SZ, especially at first onset. However studies comparing first-episode MDD and SZ are very limited.

Recent reviews of the literature indicate similar and different expression of structural and functional brain abnormalities in MDD and SZ^9^,^10^,^11^,^12^,^13^,^14^. In particular, two key brain regions involved in emotional and cognitive processing, the amygdala and prefrontal cortex (PFC), have been strongly implicated in both MDD and SZ. [In this article, the PFC is defined as the dorsal lateral PFC (DLPFC), critically engaged in cognitive regulation of emotion^15^ and working memory task^16^; the dorsal medial PFC including the dorsal anterior cingulated cortex (dACC) and its anterior section of frontal cortices– closely linked with conflict monitoring and reward processing and cognitive-motor functions^17^,^18^,^19^; and the ventral PFC (VPFC) including the orbitofrontal cortex (OFC), the inferior and rostral frontal cortices, ventral and rostral components of the ACC, mainly associated with emotional^20^ and hedonic processing^21^]. Postmortem histological studies indicated volume/cell munbers, levels of neurotransmitter receptors and gene expression in both the amygdala and PFC in MDD and SZ^22^,^23^,^24^,^25^,^26^,^27^,^28^,^29^,^30^. Structural and functional magnetic resonance imaging (MRI) studies demonstrate amygdala abnormalities in MDD and SZ with respect to size, density and activation. Previous structural MRI studies provided support MDD enlarged amygdala volume^31^,^32^ or reduced amygdala volume^33^,^34^ and decreased amygdala gray matter (GM) concentration^35^ and SZ reported decreased amygdala volume and GM density^36^,^37^,^38^. Functional MRI MDD demonstrated increased activation in the amygdala^39^,^40^,^41^,^42^ and SZ altered functional activation in amygdala^43^,^44^. Most interestingly, in the PFC, MDD and SZ appear to have some differential involvement of PFC subregions based on other MRI studies. Morphological and functional alterations have been shown primarily in the DLPFC^39^,^45^,^46^,^47^ and VPFC^46^,^48^ in MDD, though dACC deficits have also been observed^49^, whereas the majority of the MRI studies report abnormalities in the DLPFC^46^,^50^,^51^ and dACC^17^ in SZ, as well as the dorsomedial PFC (DMPFC)^52^. Connectivity studies have found abnormalities in PFC-amygdala functional connectivity (FC) in both MDD and SZ^39^,^53^,^54^,^55^,^56^, including altered FC between the DLPFC and amygdala^39^,^53^ and the VPFC and amygdala^54^,^55^ in MDD, and abnormalities in DLPFC-amygdala FC in SZ^56^.

We are not aware of any study directly comparing the resting-state FC (rsFC) between the amygdala and PFC in drug-naïve, first-episode MDD and SZ. Studies of drug naïve, first episode populations minimize confounding factors of medication effects and illness chronicity in understanding the development of psychiatric disorders. In this study, we performed a seed-based analysis of rsFC between the amygdala and PFC in first-episode, drug-naïve MDD and SZ, and healthy control (HC) participants aged 13 to 30 years. We selected this specific age range due to the critical brain changes that occurs during this period in the development of MDD and SZ^57^,^58^,^59^. Consistent with previous MRI studies in MDD or SZ, we hypothesized that (1) amygdala-PFC rsFC would be altered in the MDD and SZ groups, compared to the HC group, (2) there would be similarities in amygdala-DLPFC rsFC abnormalities between the MDD and SZ group, (3) there would be differences in amygdala to other regions of PFC rsFC abnormalities between the MDD and SZ group, for example, amygdala-VPFC rsFC abnormalities in MDD and amygdala-dACC rsFC alterations in SZ.

## Methods

### Subjects

This study totally recruited 94 drug naïve, first episode MDD and SZ aged from 13 to 30 years. Participants included 49 drug-naïve, first episode MDD (mean age 19.35 ± 6.03 years, 32 females), 45 drug-naïve, first episode SZ (mean age 18.42 ± 3.84 years, 26 females), and 50 HC individuals (mean age 18.18 ± 3.92 years, 33 females). MDD and SZ participants were recruited from the outpatient clinics at the Department of Psychiatry, First Affiliated Hospital of China Medical University, Shengyang China. HC participants were recruited from Shengyang, China using community advertisement. The presence or absence of Axis I diagnoses were independently determined by two trained psychiatrists using the Structured Clinical Interview for DSM-IV Axis I Disorders (SCID) in participants ≥18 years old and the Schedule for Affective Disorders and Schizophrenia for School-Age Children-present and Lifetime Version (K-SADS-PL) in participants younger than 18 years old. MDD and SZ participants respectively met the DSM-IV criteria for MDD or SZ without any other current or lifetime Axis I disorders, including substance abuse and dependence. Efforts were made to follow-up on subjects and confirm their diagnosis, particularly those in the early course of illness. Of the 45 subjects with SZ, 37 were within the first year of initial symptom onset and DSM-IV criteria for SZ. At one year follow-up, these subjects continued to meet DSM-IV criteria for SZ. Of the total 49 MDD subjects in this study, 44 remain as MDD at two year follow-up, and 5 remain as MDD at one year follow-up. HC participants did not have any first-degree relatives with Axis I disorders. For all three groups, individuals were excluded if any of the following were present: (1) any MRI contraindications; (2) history of head trauma with loss of consciousness 5 or more minutes or any neurological disorder; and (3) any concomitant major medical disorder. All participants were right handed and scanned within 24 hours of initial contact with the research team. All participants provided written informed consent after detailed description of the study. If participants were younger than 18 years old, participants gave written informed assent, and their parent/legal guardian provided written informed consent after receiving a detailed description of the study. The study was approved by the Institutional Review Board of China Medical University.

Symptom measures using the Hamilton Depression Rating Scale (HAMD), the Brief Psychiatric Rating Scale (BPRS) and Hamilton Anxiety Rating Scale (HAMA) and neuropsychological function using the Wisconsin Card Sorting Test (WCST) were obtained in all three groups.

### MRI data acquisition

MRI data was acquired using a GE MR Signa HDX 3.0 T MRI scanner at the First Affiliated Hospital, China Medical University, Shenyang, China. Head motion was minimized with restraining foam pads. A standard head coil was used for radio frequency transmission and reception of the nuclear magnetic resonance signal. The participants were asked to keep their eyes closed but remain awake during the scan. FMRI images were acquired using a spin echo planar imaging (EPI) sequence, parallel to the anterior–posterior commissure (AC–PC) plane with the following scan parameters: repetition time (TR) = 2000 ms; echo time (TE) = 40 ms; image matrix = 64 × 64; field of view (FOV) = 24 × 24 cm^2^; 35 contiguous slices of 3 mm and without gap; scan time 6 min 40 s.

### Data processing

The resting-state fMRI data preprocessing was carried out by using SPM8 (www.fil.ion.ucl.ac.uk/spm/software/spm8) and the Resting-State fMRI Data Analysis Toolkit (REST) (www.restfmri.net). The first 10 images were deleted, and then the data underwent further preprocessing, including slice timing correction, head motion correction, spatial normalization and smoothing. Head motion parameters were computed by estimating translation in each direction and the angular rotation about each axis for each volume. Datasets were excluded if head motion was >3 mm maximum displacement in any of the x, y or z directions or 3° of any angular motion throughout the course of the scan. To access head motion confounder, we compared the mean framewise displacement^60^ among three groups, the result showed no significant differences in head motion when comparing the three groups (*p* = 0.81). Spatial normalization was performed using a standard EPI template from the Montreal Neurological Institute (MNI). The voxel size was resampled to 3 × 3 × 3 mm^3^. Spatial smoothing was performed with an 8-mm full-width at half maximum (FWHM) Gaussian filter. Linear detrending and temporal bandpass (0.01–0.08 Hz) filtering were performed to remove low-frequency drifts and physiological high-frequency noise. Linear regression of head motion parameters, global mean signal, white matter signal and cerebrospinal fluid signal were performed to remove the effects of the nuisance covariates.

### Functional connectivity analysis

Functional connectivity analysis was performed using correlation analysis between the seed amygdala ROI and PFC mask in a voxel-wise manner using REST. The correlation coefficients were then transformed to Z-values using the Fisher r-to-z transformation. The amygdala was selected as the region-of-interest (ROI). The bilateral amygdala ROI was defined according to the automated anatomical labeling (AAL) template^61^ contained in REST, which has been resampled to 3 × 3 × 3 mm^3^. The BOLD time series of the voxels within the ROI were averaged to generate the reference time series for the ROI. A PFC mask was created using the normalized T1-weighted high-resolution images of all participants, which were skull-stripped using BrainSuite2 (http://brainsuite.usc.edu). The PFC mask included Brodmann areas (BA) 9–12, 24, 25, 32, and 44–47. Only voxels within this mask were further analyzed.

### Statistical analysis

Demographic and clinical characteristics were analyzed using IBM SPSS Statistics for Windows, Version 21.0 (Armonk, NY, USA). A one-way ANOVA was used to compare rsFC among the three groups. The contrast map threshold was set at *p* < 0.05 for each voxel with a cluster size of at least 40 voxels (1080 mm^3^), corrected for multiple comparison as determined by AlphaSim (see program AlphaSim by B.D. Ward in AFNI software. http://afni.nimh.nih.gov/pub/dist/doc/manual/AlphaSim.pdf). Z-values were extracted from the PFC regions showing significant differences among the three groups. Post hoc two-sample t-test of the Z-values between each pair group (HC vs. MDD, HC vs. SZ, MDD vs. SZ) were performed using SPSS. Statistical significance was determined by *p* < 0.05.

## Results

### Demographics, clinical characteristics and cognitive function

Demographics, clinical characteristics and cognitive function of participants are shown in Table 1. There were no significant differences in age or gender (*p* > 0.6), or education (*p* = 0.05) among the three groups. And there was also no significant difference in duration of illness between MDD and SZ groups (*p* = 0.88). Significant differences in HAMD, HAMA, and BPRS scores (*p* < 0.01) were observed among the three groups. Post-hoc analyses showed higher HAMD and HAMA scores in the MDD group compared to the SZ and HC group and in the SZ group compared to the HC group. BPRS scores were significantly higher in the SZ group when compared to the MDD and HC groups. Significant differences were also observed in WCST-correct responses, WCST-categories completed and WCST-total errors scores among the three groups (p < 0.05). Post-hoc analyses showed lower WCST-correct responses, WCST-categories completed and WCST-total errors scores in the SZ group compared to the MDD and HC group.

### Amygdala-PFC Connectivity

**Figure 1:**
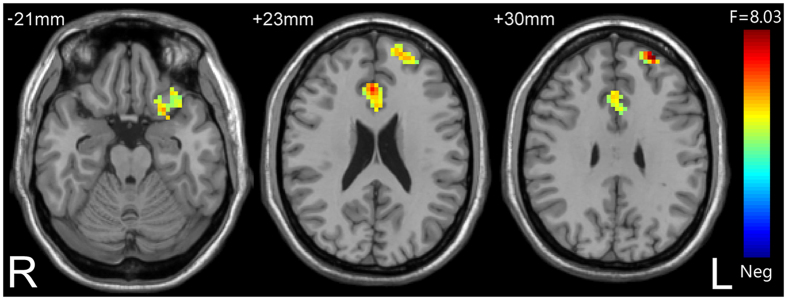
The results of one-way ANOVA showing abnormalities in amygdala-VPFC, amygdala-DLPFC and amygdala-dACC resting-state functional connectivity in the three group comparison.

There were significant differences in amygdala- VPFC, amygdala-DLPFC and amygdala- dorsal ACC (dACC) rsFC in the three group comparison (*p* < 0.05, corrected) (Table 2; Fig. 1).

**Figure 2:**
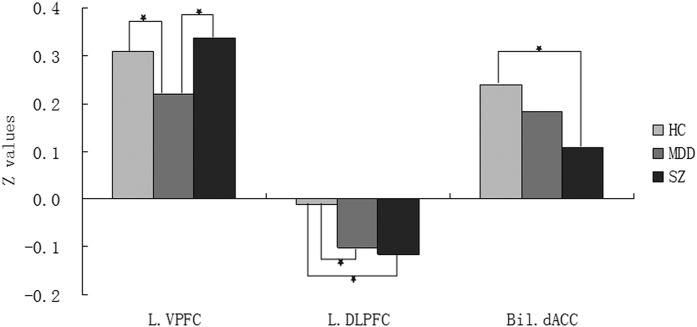
Post hoc comparison showing Z values differences at peak voxel between each pair group (HC vs. MDD, HC vs. SZ, MDD vs. SZ), *p < 0.01. HC, Healthy control; MDD, Major depressive disorder; SZ, Schizophrenia. L.VPFC, left ventral prefrontal cortex, L.DLPFC, left dorsal lateral prefrontal cortex, Bil.dACC, bilateral dorsal anterior cingulated cortex.

Post hoc pairwise comparisons found significantly decreased rsFC between the amygdala and VPFC in the MDD group, when compared with the HC and SZ groups (*p* < 0.05, corrected). No significant difference in amygdala-VPFC rsFC was observed between the HC and SZ groups. Amygdala-DLPFC rsFC was significantly decreased in the MDD and SZ groups, compared to the HC group (*p* < 0.05, corrected). Significantly decreased rsFC between the amygdala and dACC was found in the SZ group, compared to the HC group. No significant differences were shown in amygdala-dACC rsFC in the MDD group when compared to the HC and SZ groups (*p* < 0.05, corrected) (Fig. 2).

### Clinical variables

Exploratory analyses did not reveal any significant correlations between rsFC in regions showing significant group differences and HAMD, BPRS, HAMA scores, and WCST in the MDD and SZ groups.

## Discussion

As far as we are aware, this is the first study to investigate rsFC abnormalities between the amygdala and PFC in drug-naïve, first-episode adolescents and young adults with MDD and SZ. Our findings support similarities and differences in amygdala-PFC rsFC abnormalities between drug-naïve, first episode adolescents and young adults with MDD and SZ. Consistent with our previous hypotheses in this study, significant differences were found in rsFC between the amygdala and the VPFC, DLPFC, and dACC in the three group comparison (MDD, SZ, and HC). Both the MDD and SZ groups demonstrated significant decreases in amygdala-DLPFC rsFC when compared to the HC group. Differences between the MDD and SZ group were observed in amygdala-VPFC and amygdala-dACC rs FC. The MDD group had significantly decreased rsFC between the amygdala and VPFC, compared to the HC and SZ groups. Significant decrease in amygdala-dACC rsFC was found only in the SZ group when compared to the HC group. Results also suggest different effects of MDD and SZ on amygdala-dACC rsFC with the MDD group showing intermediate effects, although no significant difference in amygdala-dACC rsFC was observed between the MDD and SZ groups or the MDD and HC groups. The observed similarities and differences in amygdala-PFC rsFC may reflect neural mechanisms that underlie the clinical observations of symptom overlap during initial illness and divergence in longitudinal course in MDD and SZ. However, we did not observe any significant correlation between amygdala-PFC rsFC and symptom measures using the HAMD, BPRS, or HAMA.

Interestingly, our findings seem to reflect general conceptualization and differentiation of MDD and SZ: MDD is a disorder of primarily emotional processing and regulation whereas cognitive deficits predominate in SZ. Previous studies implicate the VPFC and amygdala as core components of the cortico-limbic circuitry and interactions in emotional processing^20^,^62^, especially in an affective disorder^54^,^55^,^56^,^63^. For example, our previous study by Liu *et al*. reported decreased rsFC between the amygdala and the VPFC in BD^56^, and our another resting-state fMRI study identified a negative correlation in VPFC-amygdala activity in BD^63^. In this study, decreased amygdala-VPFC rsFC in MDD was shown, consistent with our previous findings by Tang *et al*. and by Kong *et al*. of decreased functional connectivity amygdala-left VPFC and amygdala-left rostral PFC in treatment-naïve MDD^54^,^55^. We did not observe similar findings in the SZ group.

The dACC has been associated with various functions including conflict monitoring, reward processing and cognitive-motor functions and is also thought to be a critical component in the salience network in SZ^64^. These functions may be relevant to the development of psychotic symptoms such as delusions and hallucinations^65^. The ventral ACC is mainly activated within emotional process, whereas the dorsal ACC mostly engages in cognitive tasks^66^. For example, greater improvement in depressed bipolar adolescents was associated with baseline higher activity in ventral ACC to mild happy faces during emotion processing by fMRI^67^. The patients with schizophrenia had abnormalities in activation of the dorsal ACC in cognitive functioning by fMRI^68^. We found significantly altered amygdala-dACC rsFC in the SZ group when compared to the HC group; this was not shown in the MDD group. Our study results also suggest intermediate effects on amygdala-dACC rsFC in MDD, which would be consistent with the presence of more subtle cognitive impairments in MDD compared to SZ.

Decreased rsFC between the amygdala and DLPFC were found in both the MDD and SZ groups in this study, consistent with previous studies in MDD and SZ^39^,^53^,^56^. The DLPFC is thought to be involved in cognitive regulation of emotion, working memory, planning and cognitive flexibility^69^. It may be necessary to translate information about value into goal representations and to maintain such information so that it can be implemented as action plans to achieve the desired outcome^70^. The evidence indicated the involvement of the DLPFC in emotional processing and regulation and cognition abnormalities in the DLPFC in both MDD and SZ. For example, depressed subjects displayed decreased DLPFC activity in response to cognitive tasks^39^, and patients with SZ reduced engagement of the DLPFC within the working memory network^71^. In addition, the meta-analytic results showed lower response in the dorsolateral prefrontal cortex in individuals with major depressive disorder than in healthy subjects^72^ and the review supported physiological dysfunction of dorsolateral prefrontal cortex in schizophrenia^73^. Although the mechanism of interaction between DLPFC and amygdala remains unknown, our findings confirmed that the interaction of DLPFC-amygala could be closely associated with the pathophysiology of MDD and SZ.

Exploratory analyses did not reveal any significant relationship between rsFC in regions showing significant group differences and symptom measures and WCST scores in MDD and SZ. The reasons for this are unclear. The measures used likely may not sufficiently capture the impairments in emotional processing and cognitive function related to the rsFC alterations observed herein as previously discussed. They may be too broad and overinclusive, and more targeted and refined approaches are needed to further examine the relationship between rsFC alterations and clinical symptoms and neuropsychological function. However, our findings implicate greater severity of impairments in SZ with given greater magnitude of rsFC alteration in SZ than MDD. This is consistent with the predicted clinical course and outcome for these disorders. Future studies should include more comprehensive and refined symptom and neuropsychological measurements to further understand the relationship between brain function and clinical and cognitive manifestations.

There are limitations in this study. Educational level was marginally different among the three groups (p = 0.05), and it may have contributed to our findings. Educational level has been previously shown to impact brain function^74^. Further, as the study included subjects early in the course of illness, the results herein should be interpreted with caution. Follow-up studies have found that 20 to 40% of adolescents with MDD develop BD within a period of 5 years after the onset of depression^75^. The longitudinal diagnostic stability for these subjects remains to be seen with ongoing efforts for follow-up and diagnostic confirmation.

In summary, the findings of this study support our a prior hypotheses that there are abnormalities in amygdala-PFC rsFC in drug-naïve, first episode adolescent and young adult with MDD and SZ and that there are similarities and differences in these abnormalities between the two disorders. Both MDD and SZ appear to have similar alterations in amygdala-DLPFC rsFC. Abnormalties amygdala-VPFC rsFC may be specific to MDD whereas those in amygdala-dACC rsFC may be more related to and severe in SZ. These findings may reflect the neural mechanisms underlying the initial similarities and then divergence in clinical course in adolescent and young adult with MDD and SZ. Moreover, they may indicate potential differentiating markers at first-onset that may improve early diagnosis, intervention and treatment in adolescent and young adult with MDD and SZ. And the clinicians could treat patients in the 2 disorders using physical therapy related brain regions.

## Additional Information

**How to cite this article**: Wei, S. *et al*. Similarities and differences of functional connectivity in drug-naïve, first-episode adolescent and young adult with major depressive disorder and schizophrenia. *Sci. Rep.* **7**, 44316; doi: 10.1038/srep44316 (2017).

**Publisher's note:** Springer Nature remains neutral with regard to jurisdictional claims in published maps and institutional affiliations.

## Figures and Tables

**Table 1:** Demographics, clinical characteristics and cognitive function of participants.

Number (n)	MDD	SZ	HC		*p*
	49	45	50		
**Demographic characteristic**					
Gender (male/female)	17/32	19/26	17/33	*χ*^*2*^ = 0.83	0.66
Age (years), mean ± SD	19.35 ± 6.03	18.42 ± 3.84	18.18 ± 3.92	*F* = 0.46	0.63
Education (years), mean ± SD	12.12 ± 3.77	10.71 ± 2.43	11.92 ± 2.60	*F* = 2.94	0.05
**Clinical characteristic**					
HAMD score, mean ± SD	20.86 ± 8.70	9.52 ± 11.41	1.32 ± 1.65	*F* = 78.64	0.000
BPRS score, mean ± SD	23.08 ± 8.20	40.00 ± 13.03	18.26 ± 0.86	*F* = 38.39	0.000
HAMA score, mean ± SD	17.14 ± 8.81	6.93 ± 8.45	0.78 ± 2.16	*F* = 62.35	0.000
Duration of illness (months), mean ± SD	4.59 ± 6.28	4.32 ± 10.46	—	*T* = *0.158*	0.88
**Cognitive function**					
WCST	(n = 28)	(n = 29)	(n = 33)		
correct responses	26.21 ± 10.78	19.52 ± 12.90	28.61 ± 12.80	*F* = 4.48	0.01
categories completed	2.89 ± 1.75	1.72 ± 1.89	3.70 ± 2.08	*F* = 8.17	0.001
Total errors	21.79 ± 10.78	28.55 ± 13.01	19.39 ± 12.80	*F* = 4.52	0.01
Perseverative errors	8.75 ± 7.27	14.10 ± 14.05	8.79 ± 9.54	*F* = 2.47	0.09
Non-perseverative errors	13.04 ± 5.19	14.38 ± 8.50	10.61 ± 5.12	*F* = 2.77	0.07

MDD, Major depressive disorder; SZ, Schizophrenia; HC, Healthy controls; SD, Standard Deviation; HAMD, Hamilton Depression Rating Scale; BPRS, Brief Psychiatric Rating Scale; HAMA, Hamilton Anxiety Rating Scale, WCST, Wisconsin Card Sorting Test.

**Table 2:** Brain regions showing significant changes in the bilateral amygdala functional connectivity.

Cortical Regions (Brodmann Areas)	MNI Coordinates				
	Cluster Size	X	Y	Z	*T* values^a^
Left ventral prefrontal cortex (47)	58	−24	15	−21	5.30
Left dorsal lateral prefrontal cortex (9/10)	76	−27	54	30	8.03
Bilateral dorsal anterior cingulate cortex (24/32)	165	6	12	39	7.45

^a^These findings correspond to a corrected *P* < 0.05 by AlphaSim correction.
